# Disciplinary behaviour management strategies in schools and their impact on student psychosocial outcomes: A systematic review

**DOI:** 10.3310/nihropenres.13563.2

**Published:** 2024-07-01

**Authors:** Sharea Ijaz, James Nobles, Loubaba Mamluk, Sarah Dawson, Bonnie Curran, Rachael Pryor, Sabi Redwood, Jelena Savović

**Affiliations:** 1 Population health sciences, NIHR ARC West, University of Bristol, Bristol, BS1 2NT, UK; 2 School of health, Leeds Beckett University, Leeds, England, LS1 3HE, UK; 3 Public Health, Bristol City Council, Bristol, England, BS1 5TR, UK; 4 University of the West of England, Bristol, England, BS16 1QY, UK

**Keywords:** adolescent, school discipline, behaviour management, mental health, wellbeing, systematic review

## Abstract

**Background:**

Disciplinary behaviour management strategies are implemented in schools to manage pupil behaviour. There is limited evidence of their intended impact on behaviour but there is growing concern around the potential negative impacts on pupil wellbeing.

**Methods:**

We carried out a systematic review to examine the impact of these strategies on psychosocial outcomes in pupils (PROSPERO Registration: CRD42021285427). We searched multiple sources and double-screened titles, abstracts, and full texts. Data extraction and risk of bias assessment were done by one reviewer and checked by another. Results were narratively synthesised.

**Results:**

We included 14 studies, from 5375 citations, assessing temporary suspension (n=10), verbal reprimand (n=2), and mixed strategies (n=2). Depression was the most common outcome (n=7), followed by academic grades (n=4) and behaviour in class (n=4). All except one study were at high risk of bias. We found a recurring pattern in the evidence of disciplinary strategies associated with poor mental wellbeing and behaviour in pupils. The effect on academic attainment was unclear.

**Conclusions:**

Disciplinary behaviour management strategies may have negative impact on pupil mental wellbeing and class behaviour. These important consequences should be assessed in better designed studies before these strategies are implemented.

## Plain English Summary

How does school discipline affect pupil mental health and wellbeing? A systematic review

In England, a variety of approaches are used in schools to manage pupils’ behaviour. There isn’t much evidence about their impact on behaviour, but there’s growing concern they might negatively affect pupils’ wellbeing.

We systematically reviewed the published research to understand the impact of these behaviour management strategies on pupils’ wellbeing. We searched multiple sources and two people looked at titles, abstracts and full papers.

One reviewer extracted the data and assessed the studies for risk of bias, while another checked this work. Results from all studies were combined together in text and tables.

We included 14 studies from the 5,375 papers that we looked at. Included studies explored at a range of strategies, including temporary suspension (10 studies), verbal reprimand or being told off (2 studies), and a combination of strategies (2 studies).

Seven studies looked at whether these strategies led to depression, four looked at the impact on exam grades and four on behaviour in class. All except one study were at high risk of bias, meaning we can’t fully trust their findings.

Many of the studies showed these disciplinary strategies were linked to poor mental wellbeing and behaviour in pupils. The effect on exam results wasn’t clear.

It appears pupil mental wellbeing and class behaviour was made worse by these strategies. These are important impacts and should be researched in high quality studies before these strategies are used further.

## Introduction

Disciplinary behaviour management strategies are implemented in schools to help manage pupil behaviour. There are several approaches towards behaviour management. There are punitive strategies which align with the theory of assertive discipline ^1^, which set out clear rules that reward good behaviour and punish poor behaviour. Punitive approaches will typically directly respond to poor behaviour, whereby punishing a pupil is anticipated to reduce the likelihood of repeated disruptive behaviour. However, if a pupil misbehaves again or dependent on the misbehaviour itself, increasingly severe forms of punishment are then used. Punitive approaches include strategies such as verbal reprimanding (e.g., being shouted at in class), detentions, isolation rooms, in- and out-of-school suspensions, and expulsion (permanent exclusion). These punitive approaches are commonplace in the western world ^2, 3^, and are more common in secondary schools in UK ^4, 5^.

Conversely, there are alternative approaches that aim to understand why pupils act as they do, in the context of poor behaviour, and then work with the child to reduce the likelihood of these behaviours recurring. These include restorative approaches ^6^, trauma informed approaches ^7^, collaborative problem solving ^8^, positive behavioural intervention and support (PBIS) ^9^, or attachment-based strategies ^10^. These strategies support pro-social behaviour between pupils, and collaborative interaction between pupils and teachers ^11^. The evidence base in support of these alternative approaches has developed in recent years ^12– 14^.

There is limited evidence regarding the intended impact of punitive approaches on behaviour and academic outcomes for affected pupils and their peers ^15– 18^. There is also a growing concern for the potential negative implications that punitive approaches may have on wellbeing outcomes later in life ^19, 20^. This is important given that young people’s mental health has declined in recent years in the UK, partly due to disruptions in school and home routines following COVID-19 and the pandemic response strategies ^21^.

This concern about the potential negative mental health impact on pupils was voiced by secondary school age young people in a public consultation meeting in Bristol (England) when collaboratively identifying research priorities.

This review was then developed with input from these young people to investigate the existing evidence on the effects of punitive behaviour management strategies on mental health and wellbeing in secondary school age children and young people ^22^.

## Methods

### Objectives

To examine whether the use of disciplinary behaviour management strategies (interventions) in secondary schools leads to adverse psychosocial outcomes for pupils

Secondary objectives were:

- To explore whether adverse effects differ between children of different socio-demographic backgrounds
- To determine whether there is evidence of effectiveness for these disciplinary behaviour management strategies in improving behaviour and academic outcomes

This review was registered with the international Prospective Register of Systematic Reviews (PROSPERO) in October 2021. Registration number CRD42021285427 ^23^.

***Public Involvement***

We held three involvement sessions with young people aged between 11 and 16 years old to develop the broad research questions. The first session involved the Young People’s Advisory Group (YPAG; a local public involvement group for young people interested in research) raising concerns about the effect of disciplinary behaviour management strategies on pupil wellbeing. A second workshop was conducted with funding from Create to Collaborate ^24^ to explore these concerns with a broader group of young people affiliated with a mental health charity. In this workshop, young people suggested that some school discipline practices affect their wellbeing negatively. We then ran a third workshop to refine the review questions and search terms with the input from YPAG.

***Eligibility criteria***

We included randomised and non-randomised study designs (including longitudinal and cross-sectional surveys). We excluded solely qualitative studies as this review’s scope was limited to effects of interventions.

Based on our public involvement work with young people, we were interested in zero-tolerance, punishment-based, or punitive disciplinary strategies that include verbal reprimanding, behaviour monitoring and reporting, isolation, detentions (either during- or after- school hours), and suspension (inclusive of temporary- or fixed- term exclusion). We did not include studies which only focused on permanent exclusion or expulsions from schools.

We limited our inclusions to the UK and other high-income countries in The Organisation for Economic Co-operation and Development (OECD). This meant that approaches such as corporal punishment, and physical- or chemical- restraint were not included given that they are not implemented in a UK (or similar) context.

We included studies of children and young people from the general population, aged 11–16 years, attending a main-stream school. We excluded studies focused on pupils in specialist schools, such as secure centres for children (similar to a juvenile correction facility in the USA), special behavioural units, and Special Educational Needs and Disability (SEND) schools.

Our primary outcomes were any measures of mental health and wellbeing. We included academic and social outcomes as secondary outcomes.

***Search strategy***

We developed search strategies with an information specialist (SD) and searched seven online databases from inception to October 15th, 2021: MEDLINE; Embase; PsycINFO; British Education Index; Australian Education Index; Education Resources Information Centre (ERIC); Web of Science Social Science Citation Index (SSCI). See *Extended data* for search strategies ^25^. We also sent a standardised email through the Children and Young People’s Mental Health Coalition ^26^ to their 237 member organisations to help identify grey literature.

***Study selection***

Titles and abstracts identified through electronic database- and web- searching were independently screened for relevance in duplicate (JN, SI, & LM) using Rayyan ^27^. Full texts were then retrieved for all relevant references and assessed against the inclusion criteria, in duplicate. Reasons for exclusion were documented (see table S1 in supplementary file) at this stage. Any discrepancies between reviewers at either stage were resolved through discussion or via a third reviewer (JS).

***Data extraction and risk of bias assessment***

Data extraction was undertaken by one reviewer (JN) using a standardised form in Microsoft Excel. To minimise bias and errors, a second reviewer (SI) checked the data extracted from all included papers. We extracted information on the following: a) study design, b) sample size and characteristics, c) the behaviour management strategy being studied, d) control / comparator [where available], e) context and setting, and f) information about, and results pertaining to, the primary and secondary outcomes. We assessed risk of bias in included studies using Cochrane Effective Practice and Organisation of Care (EPOC) group’s criteria for nonrandomised studies ^28^. We considered a study to be at an overall low risk of bias when all items were scored at low risk, at an overall moderate risk of bias when more than half the items were at low risk of bias, and all others were rated high risk of bias.

***Synthesis***

We planned for a random effects meta-analysis if combinable data were available. However, a meta-analysis was not conducted as the studies were highly heterogeneous across populations, comparisons, follow up times, outcome measures, effect measures, and notably, study designs. The analyses presented were often unadjusted with numbers of analysed participants unclear. A narrative synthesis was therefore performed. Pooling these disparate data from high risk of bias studies in a meta-analysis would not have changed our conclusions and recommendations.

## Results

### Study selection

**Figure 1.**
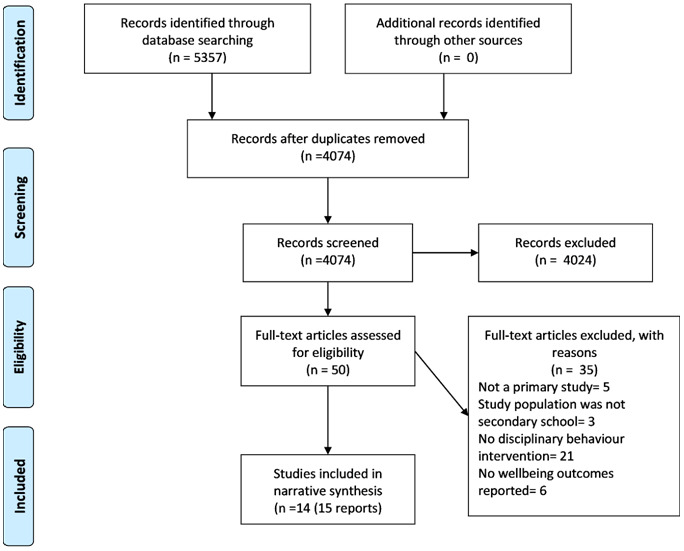
PRISMA Flow diagram of review process.

Electronic searches resulted in 5357 citations. We found no additional studies through contacts with experts and third sector organisations. Fifty papers were included for full text assessment. After full text screening, 14 studies ^29– 42^ out of these 50 were included in narrative synthesis. See Figure 1 for detail of the process.

### Description of included studies

Ten studies were from USA, followed by two from Australia and the UK respectively, and one from Poland. We found no randomised trials. Studies were either surveys or uncontrolled before and after designs. Sample sizes across studies varied widely, ranging from 23 pupils to 33 572 pupils (median = 1811 pupils).

Studies typically included more females than males. Most studies (n=9) ^29, 30, 32, 33, 35, 37– 40^ included high school pupils. White pupils were included less often (mean 36%, median 30% across studies) than non-white pupils. Five studies ^31, 35, 39, 41, 42^ focused on deprived populations, reported as majority (>50%) children being on free school meals and/or low earning.

Temporary suspension from school was most frequently studied (n=11) ^29, 31, 33– 35, 37, 39– 43^, followed by verbal reprimanding or punishment (n=2) ^36, 38^ and various mixed (multiple combined) strategies (n=2) ^30, 32^. We present result for these categories separately below.

The most common studied outcome was depression (n=7) ^30– 32, 34, 35, 37, 40^ using various scales: PHQ-9 (Patient health Questionnaire); CESD (Center for Epidemiological Studies Depression); SDQ (Strengths and Difficulties Questionnaire); California Healthy Kids Survey (WestEd) (Depression subscale); and Add Health survey. Two studies assessed internalising symptoms (i.e., problems of withdrawal, somatic complaints, and anxiety/depression) ^44^, one using Teacher observation of classroom adaptation checklist (TOCAC) ^31^ and Youth Self Report (YSR) in the other ^41^. One study assessed externalising symptoms (i.e., problems of aggression, impulsivity, and inattention) ^44^ on adapted Behavior Assessment System for Children: second edition (BASC 2) scale ^29^. Anxiety was assessed in one study ^36^ using a Polish version of State-Trait Anxiety Inventory for Children. General mental wellbeing was assessed in three studies ^33, 38, 39^, one using the Warwick and Edinburgh Mental Wellbeing Scale ^33^, and two using author developed scales ^38, 39^. Five studies ^30, 35, 36, 39, 42^ assessed impact of disciplinary strategies on educational attainment. See Table 1 for details of included studies.

**Table 1.** Characteristics of included studies by disciplinary strategies.

Author (Year) Country	Study design	Disciplinary intervention	Setting, sample and demographics	Outcomes reported	Findings
**Suspension** **Strategies**					
Bottiani *et al.* (2017) ^29^ USA	Cross-sectional survey	Out-of-school suspension	*Setting*: High school *Sample size*: 19 726 pupils *Age*: 15.9 years *Sex*: 50% female *Ethnicity*: 64% White American *Deprivation*: 37.5% of *schools* provided FSM	Externalising symptoms; social belonging, perceived equity	Higher Black-to-White suspension gaps (higher suspension for Black students) led to higher levels of adjustment problems (externalising symptoms) in black pupils (γ=0.77, *p*<.001) at one year post suspension
Cohen *et al.* (2020) ^31^ USA	Prospective follow up	In-school and out-of-school suspension	*Setting*: Middle school *Sample size*: 788 pupils *Age*: N/R *Sex*: 50% female *Ethnicity*: 74% African American *Deprivation*: 75% FSM	Student-reported depression, and emotional dysregulation; behaviour; concentration in class	Neither in school nor out of school suspension frequency affected self-reported depression on PhQ-9: In school suspension effect: b=0.015; SE 0.103, standardized beta weight (95% CI) = 0.007 (−0.086, 0.100) out of school suspension effect: b=0.211 SE 0.186, standardized beta weight (95% CI) = 0.043 (−0.032, 0.118) The frequency of in- and out-of-school suspension predicted greater disruptive behaviour ( *b* = 0.119, *p* ≤ .001)) more concentration problems (b=0.118, 0.028), *p* ≤ .001). Suspension not our outcome
Fazel *et al.* (2021) ^33^ UK	Cross-sectional survey	Out-of-school suspension	*Setting*: High school *Sample size*: 1648 pupils *Age*: N/R *Sex*: 60% female *Ethnicity*: N/R *Deprivation*: N/R	Mental wellbeing; accessing mental health services	No significant difference in wellbeing scores between those who were suspended ( *n*=93) or not ( *n*=1555). 25.8% of those suspended accessed mental health services compared to 14% who were not suspended. Suspended pupils more likely to be male, identify as “aggressive and violent”, and have been recently bullied compared to non-suspended pupils.
Ford *et al*. (2018) ^34^ UK	Longitudinal survey	Out-of-school suspension	*Setting*: N/A *Sample size*: 5326 pupils *Age*: N/R *Sex*: N/R *Ethnicity*: N/R *Deprivation*: N/R	Depressive symptoms; psychopathology	At baseline, suspended pupils have 13.7 (95%CI: 10.8-17.4) times greater odds of having a psychiatric disorder compared to non-suspended. At follow up, odds were 5.5 times greater (95%CI: 3.6-8.4). Suspended pupils, have poorer scores on the Strengths and Difficulties Questionnaire at follow up compared to non-suspended (OR: 6.8, 95%CI: 5.9-7.7) and higher odds of a new psychiatric disorder (OR: 7.1, 95%CI: 5.1-9.9). Not our outcome- the effect of these covariates (subgroups should be on our outcomes not on exposure)
Gase *et al*. (2017) ^35^ USA	Cross-sectional survey	Out-of-school suspension	*Setting*: High school *Sample size*: 33 572 pupils *Age*: N/R *Sex*: 50.2% female *Ethnicity*: 75% Hispanic *Deprivation*: 70% of *schools* provided FSM	Depressive symptoms or suicidal ideation; educational attainment; health behaviour	Suspension rates not associated with depressive symptoms or suicidal ideation (OR: 0.39, 95% CI: 0.15-1.04) Suspension rate was significantly associated with marijuana use (adjusted odds ratio 6.41, 95% CI 1.64, 25.07) but not tobacco use or alcohol use. No significant effect on grade point average in multilinear regression: −0.04 (−0.60, 0.53), and after controlling for age, sex, ethnicity and SES: 0.02 (−0.57, 0.62).
Quin (2019) ^37^ Australia	Cross-sectional survey	Out-of-school suspension	*Setting*: High school *Sample size*: 304 pupils *Age*: 14.7 years *Sex*: 45.4% female *Ethnicity*: 44% Asian, 24% English Australian *Deprivation*: 28.9% received Education Maintenance Allowance	Depressive symptoms; school belonging; behaviour	39% of suspended students had a borderline score on the Strengths and Difficulties Questionnaire compared to 19% of non-suspended students ( *p*<0.001). Statistically significant relationship between the total difficulties scores and suspension: χ2(1) = 12.35, p < .001
Rose *et al.* (2017) ^39^ USA	Cross-sectional survey	Out-of-school suspension	*Setting*: High school *Sample size*: 1170 pupils *Age*: 15.0 years *Sex*: 52% female *Ethnicity*: 69.2% African American *Deprivation*: 54.8% of families earned less than $32 000	Mental wellbeing (poor, content or positive); educational attainment	Suspended pupils had 2.1 times greater odds of being in poor mental health group rather than positive mental health group ( *p*≤0.05). Similarly, they had 1.9 times greater odds of being held back a grade ( *p*≤0.05). Findings on relationship between grades and suspension not reported. No statistically significant effect of ethnicity when comparing the positive mental health group to the others. But males had higher odds (OR=1.865) of being in the troubled group, and pupils from higher-income families had lower odds of being in vulnerable group (OR=0.721)
Rushton *et al.* (2002) ^40^ USA	Longitudinal survey	Out-of-school suspension	*Setting*: High school *Sample size*: 13 568 pupils *Age*: 15.6 years *Sex*: 49.7% female *Ethnicity*: 68% White American *Deprivation*: 11.5% received public assistance	Depressive symptoms	Suspended pupils had 1.9 (95%CI: 1.3-2.7) times greater odds of having depressive symptoms compared to non-suspended pupils. Results adjusted for race, grade in school, socio-economic status, maternal educational status, and single-parent household.
Smokowski *et al.* (2014) ^41^ USA	Longitudinal survey	Out-of-school suspension	*Setting*: Middle school *Sample size*: 4229 pupils *Age*: 12.8 years *Sex*: 52% female *Ethnicity*: 28.4% American Indian, 26.7% White American, 23.3% African American *Deprivation*: 85.8% FSM	Internalising symptoms; Self-esteem	For every out-of-school suspension a pupil receives, their odds of internalised symptomology increased by 5% ( *p*≤0.05). No association between out-of-school suspension and self-esteem.
Stanley *et al.* (2006) ^42^ USA	Cross-sectional survey	Out-of-school suspension	*Setting*: Middle school *Sample size*: 23 pupils *Age*: N/R *Sex*: 35% female *Ethnicity*: 69% Hispanic *Deprivation*: 73% FSM	Adjustment problems,); Student academic resources (composite score of academic performance, academic habits, parent participation, social skills) Behavioural problems	Suspension was significantly associated with greater adjustment problems scores ranging from 56 to 70 when normal scores are below 50 Suspension was also associated with significantly lower t scores on academic resources ranging from 26 to 36 which are below the norm (50 and above)
Verbal reprimand strategies					
Piekarska (2000) ^36^ Poland	Prospective cross-sectional survey	Verbal punishment induced school stress from teachers, inclusive of: 1) Threats 2) Mockery 3) Humiliation 4) Insulting 5) Verbal attack 6) Written or oral tests	*Setting*: Primary school in Poland (includes grade 1-8) Sample size: 271 pupils *Age*: 13-14 years *Sex*: N/R *Ethnicity*: N/R *Deprivation*: N/R	Anxiety; Educational attainment	School stress due to poor performance on sudden written and oral tests, reported by 77% and 53% of pupils respectively. Significant association between school stress and anxiety (r = 0.30, *p*<0.001). Significant association between school stress and educational attainment - grade point average (r = -0.29, *p*<0.001).
Roache *et al.* (2011) ^38^ Australia	Prospective cross-sectional survey	Verbal punishments, inclusive of yelling in anger and embarrassing students deliberately Compared to techniques using combination of rewards and punishments	*Setting*: High school *Sample size*: 1975 pupils *Age*: N/R *Sex*: N/R *Ethnicity*: N/R *Deprivation*: N/R	General wellbeing; School connectedness; Misbehaviour; Attitude and interest in subjects	No relationships reported between punishment-based or aggression-based classroom management and pupil wellbeing. Significant relationships between aggressive classroom management techniques and pupil misbehaviour (r=0.48, *p*≤0.05), being distracted from work (r=0.72, *p*≤0.05), and pupil interest in the subject (r=-0.58, *p*≤0.05).
Mixed strategies					
Chen *et al.* (2021) ^30^ USA	Prospective follow up	Total punishment, inclusive of: 1) Corporal punishment 2) In-school and out-of-school suspension 3) Expulsion 4) Referral to law enforcement 5) School-related arrests	*Setting*: High school *Sample size*: 261 pupils *Age*: 11.2 years *Sex*: 34.9% female *Ethnicity*: 100% African American *Deprivation*: 42.3% lived in relative poverty	Depressive symptoms; academic orientation; Educational attainment	Attending a school that disproportionately punished Black students predicted more depressive symptoms at age 27 years β= 0.11 (95% CI: 0.04, 0.18). Significant positive interaction between academic orientation and disproportionate school punishment β=0.12 (95% CI: 0.01, 0.24). Disproportionate school punishment did not affect educational achievement at age 27 β=0.785 (95%CI: 1.466, 3.035) however, A positive main effect of academic orientation on adult educational attainment qualified by a significant interaction with disproportionate school punishment β= 0.11 (95% CI: 0.00, 0.23).
Eyllon *et al.* (2022) ^32^ USA	Longitudinal survey	Severity of school disciplinary policies: 1) Lenient policies 2) In-school suspension 3) Out-of-school suspension 4) Expulsion Assessed on a 7-point Likert scale to produce a mean score per school	*Setting*: High school *Sample size*: 8878 pupils *Age*: 15.7 years *Sex*: 54% female *Ethnicity*: 59% White American *Deprivation*: 23% received public assistance	Depressive symptoms	Significant association between mean disciplinary policy severity and depressive symptoms. A one-unit increase in mean discipline policy severity was associated with a 1.03 unit increase in depressive symptoms among non-excluded students (95% CI: 0.15, 1.91). Controlling for ethnicity did not modify the results.

CI- confidence intervals; FSM- free school meals N/R- not reported OR- Odds ratio PHQ-9- Patient Health Questionnaire -9 SES- socioeconomic status

### Risk of bias in included studies

**Figure 2.**
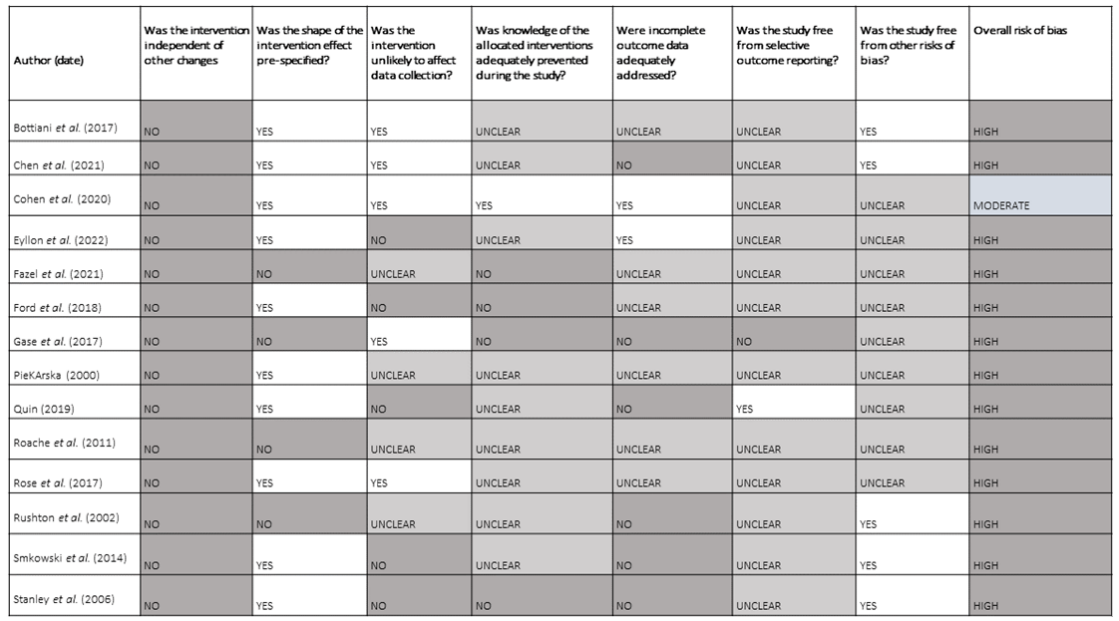
Risk of bias in included studies in the review.

Studies were mostly at high risk of bias across all domains (see Figure 2). Only one study ^31^ was considered at an overall moderate risk of bias. None were considered at a low risk of bias. For most studies there is risk of bias due to confounding. For cross-sectional studies and surveys the risk of reverse causality is a key problem, i.e., we can’t be sure whether poor mental health was the cause of 'bad behaviour' and thus the reprimand or suspension, rather than the consequence.

### Effect of disciplinary strategies on pupil mental health and wellbeing outcomes

• ***Depression***

i.   School Suspension Strategies

Five studies ^31, 34, 35, 37, 40^ reported on depression due to suspension.

Odds of greater total difficulties (SDQ) were found to be significantly higher for those who were suspended in two studies ^34, 37^. Rushton *et al*. ^40^ also found that being suspended from school was associated with increased odds of depressive symptomology. Two studies ^31, 35^ found self-reported depressive symptoms were not related to suspension.

ii.   Verbal reprimand strategies

None of the two studies assessing verbal punishment strategies reported on depression.

iii.   Mixed strategies

Two studies ^30, 32^ reported depression assessing two slightly different strategies that included suspensions and some other forms of punishments together. Both found that punishment-based policies led to more depressive symptoms, but at different time points.

Chen *et al*. ^30^ found that Black pupils attending a school that disproportionately punished Black students had greater depressive symptoms as an adult ten years later (β= 0.11,95% CI: 0.04, 0.18), compared to their White peers. Eyllon *et al*. ^32^ found strict (vs lenient) policies to increase depression in pupils so that each unit increase in school’s policy being strict led to a 1.03 unit rise in pupil depression scores on average (95% CI: 0.15, 1.91).

*Impact on population subgroups*

The two studies reporting data on ethnicity were not in agreement. While Chen *et al*. ^30^ found Black pupils to be disproportionately affected by disciplinary punishments and the consequent depression in later age, Eyllon *et al*. ^32^ found no link between ethnicity and higher depression due to strict school policies within one year.

• ***Anxiety***

No suspension or mixed strategy studies reported this outcome.

A single study set in Poland ^36^ found that higher school stress brought on by verbal reprimand strategies led to higher anxiety in pupils (R = 0.30, p <.001).

• ***Psychiatric disorder***

A single study ^34^ found children who had been suspended from school had higher odds of diagnosis of a new psychiatric disorder (OR 7.09; 95%CI 5.07 to 9.91; p < 0.001) compared to those not suspended.

No studies of verbal reprimand or mixed strategies reported this outcome.

• ***General mental wellbeing***

One study ^33^ assessing the effect of suspension found a non-significant (p=0.15) lower wellbeing (on Warwick Edinburgh Mental Wellbeing Scale) and significantly (p=0.003) greater use of mental health services for suspended children compared to pupils who have never been suspended. Another found children who were suspended were twice as likely to have poor mental health ^39^.

A single study ^38^ assessing link between verbal disciplinary strategies and pupil mental wellbeing (on author developed scale) reported no outcome data.

No study on mixed strategies reported this outcome.

• ***Internalising symptoms***

One study on suspension ^41^ found that each additional suspension per school led to increase in internalising scores by 0.05%, while another ^31^ found that suspensions were not associated with internalising problems.

No verbal reprimand or mixed strategy studies reported this outcome.

• ***Externalising symptoms***

A single study found that in schools which suspended proportionally more Black pupils than White pupils, Black pupils overall showed higher externalising symptoms ^29^.

No verbal reprimand or mixed strategy studies reported this outcome.

### Effect of disciplinary strategies on pupil social and behavioural outcomes

i.   School Suspension Strategies

Three studies ^31, 37, 42^ found suspension was associated with poorer (more disruptive, less pro-social) behaviour.

Two studies found suspensions were associated with lower perception of social belonging at school ^29, 37^.

School-level Black–White suspension gaps (i.e., excess risk of out-of-school suspension among Black students relative to White students,) were associated with Black students’ perceptions of less school equity in a single study ^29^.

A single study ^35^ found suspensions led to greater marijuana use but had no association with tobacco or alcohol use. A single study ^41^ found no association between out-of-school suspension and self-esteem.

ii.   Verbal reprimand strategies

A single study (Roache) found that aggressive verbal punishments from teachers led to increased disruptive behaviour in the classroom (r=0.48, p<0.05), being more distracted from class work (r=0.72, p<0.05), and reduced pupil interest in the subject being taught (r=-0.58, p<0.05).

No studies in this category reported social outcomes.

iii.   Mixed strategies

      No studies of mixed strategies reported social outcomes.

### Effect of disciplinary strategies on academic outcomes

i. School Suspension Strategies

Of the three studies assessing educational outcomes, one comprehensively reported data and ^35^ found no link between suspension and grade scores. This effect remained non-significant (although direction was opposite) after adjusting for demographic factors including ethnicity. One study ^39^ did not report data on the effect of suspension on grades, and the other ^42^ said they found lower scores on a composite of academic performance habits and skills but did not report data to support this finding.

ii. Verbal reprimand strategies

Piekarska *et al*. 36 found that verbal punishments from teachers caused school stress which negatively impacted academic performance as grade point average.

iii. Mixed strategies

While Chen *et al*. (2021) reported no direct effect of greater punishment on long-term educational attainment, they did find that for children who were not academically oriented, greater punishment was associated with lower educational attainment. This study included only Black American pupils.

## Discussion

### Summary of findings

Our review illustrates that evidence on the impact of disciplinary strategies in schools is scarce and of low quality. Although at high risk of bias, five out of seven studies assessing depressive symptomatology found it to be associated with exposure to disciplinary strategies. All three studies on general mental wellbeing found it to be associated with exposure to disciplinary strategies. Single studies on anxiety, psychiatric disorder diagnosis, and externalising symptoms also found that disciplinary strategies were associated with these issues. Internalising symptoms, and a similar link with externalising symptoms, were only seen in one of the two studies to be associated with a disciplinary approach. Similar effect was seen with social outcomes where, overall, disciplinary strategies were associated with poor social behaviour (n=4), lower school belonging (n=2), and greater marijuana use (n=1), but had no association with tobacco use or self-esteem (n=1). Evidence of the impact on educational attainment was limited and it was not clear how they were related to disciplinary strategies. A recent qualitative synthesis has found some evidence of a link between punitive disciplinary strategies and poor academic outcomes ^45^.

## Comparison to other systematic reviews

While there are reviews on suspensions and exclusions as outcomes ^2, 46^, we did not find any that examine the mental health or wellbeing impact of these strategies. We found one systematic review reporting that pupils experiencing exclusionary discipline were more likely to have subsequent contact with the justice system ^47^. To our knowledge, our systematic review is the first to question the impact of these strategies on mental wellbeing of school children. Considering the increasing levels of mental health problems in young people in the UK ^48^ it is important to assess these strategies for their potential impact on these outcomes which are important to pupils, their families and society.

Most of the evidence available was on suspensions. Suspensions have been rising in recent years in the UK, with the main reason for suspensions being disruptive behaviour ^49^. Our review shows that suspensions can potentially increase disruptive behaviour, thus creating a vicious cycle of increase in both.

Although not the main focus of our review, we did see across three studies that children of Black, Asian, and Minority Ethnicities (BAME) origin were often at higher risk of disciplinary actions from teachers. This is in line with recent findings from both USA ^50^ and the UK ^49^, indicating that the interaction of race and adverse childhood experiences predispose students of colour to be subject to school discipline. Future research should explore these links, and schools should consider these potential equality risks when implementing disciplinary strategies. Governments place importance on the safeguarding of all pupils’ wellbeing in their expectations from teachers ^51, 52^. Our review suggests that currently approved strategies can negatively impact student wellbeing, which can make it hard for teachers to fulfil these expectations. This can be remedied by enabling teachers use of evidence-based interventions that can reliably support pupil wellbeing. There is growing evidence on trauma informed and restorative approaches for improving and managing disruptive behaviours in schools ^53, 54^ that are less likely to negatively impact pupil wellbeing.

### Limitations of our review

We followed PRISMA standards when reporting the review and searched comprehensively using relevant scientific databases and grey literature sources. We were inclusive in our criteria for studies to allow us to examine the full range of effects of these commonly used strategies. Considering how widespread their use is, the empirical evidence on these strategies is limited for wellbeing, behaviour or academic outcomes. We found no studies from grey literature. Some of the databases we searched would have contained certain types of grey literature (e.g. conference abstracts, theses and dissertations). However, had we searched repositories of grey literature (e.g. OpenGrey or Overton (policy documents)), or the websites of international and regional education authorities, and government departments associated with our topic, we may have identified additional research published as monographs, reports, policy documents etc. It was difficult to translate our detailed search across to these grey literature sources, and we thought it more practical to talk to experts considering our limited resources.

Included studies were at high risk of bias in most domains. This is a major limitation of our findings. There is a need for better quality research to address these questions.

We searched for, and included studies that reported at least one primary (mental wellbeing) outcome. Our restriction to primary outcome reporting has likely overlooked evidence on educational and behavioural outcomes reported in studies without a focus on general mental wellbeing. Thus, our findings on these outcomes are likely not to be comprehensive, although they may be indicative of the general trend. This review included studies conducted only in mainstream schools and therefore the findings do not extend to other settings. However, when screening the literature, there were studies that focused on specialist schools or exclusively including pupils with additional learning needs (e.g., attention deficit hyperactivity disorder) and these should be assessed in a separate review.

We included all author definitions for strategies (e.g., suspension or temporary exclusion could be anything from a few hours to several days, and may or may not include supervised confinement to a room or location in school) to not miss any relevant evidence. There is however a lack of clear definitions and descriptions for any of the disciplinary strategies. Thus, there is a need to clearly define these interventions and their proposed impact before research on these can give clear conclusion on their relative impacts. For example, UK defines suspensions as any fixed time exclusion between one school period (length of a lesson varies from 30 minutes to 120 minutes) and 45 school days ^49^. This definition may be different from those used in other nations. This would invariably also be reflected in studies from different countries. We would anticipate that the effect of a 45-minute isolation may be different from that of a week-long or month-long suspension. A differential or dose response effect may only be elicited if the definitions used in each study are clear.

## Conclusions

Existing evidence indicates that disciplinary behaviour strategies might lead to poorer mental wellbeing and behaviours for pupils. There is some evidence to suggest these strategies may also inadvertently increase inequalities. However, the limitations of quality and size of the evidence precludes clear conclusions.

This means schools, and decision makers within educational systems, need to be cautious when adopting and advocating these strategies until better evidence on these is available. It would also be advantageous for schools to share data on disciplinary strategies and pupil health outcomes with research teams to facilitate a deeper level of exploration.

There is a need to assess wellbeing, social and academic effects of these disciplinary strategies (and other strategies) ideally in robustly designed trials comparing school clusters with different strategies in place. These trials (natural experiments) should be complemented with qualitative exploration of pupil perceptions of these strategies and their outcomes in various contexts. There are county-wide surveys and school-based surveys in the UK that routinely measure the health and wellbeing of pupils. These data could be compared to respective school level suspension rates and other disciplinary strategies (e.g., isolation/isolation rooms). As these wellbeing surveys are repeated annually, we should also be able to see trends of wellbeing over time, as well as the potential impacts of changing national or regional disciplinary policies on these outcomes at school level. Follow up data should also be gathered beyond the school period (into adulthood) as disciplinary strategies may have long term consequences ^30, 47^.

Disciplinary strategies aimed at improving behaviour at school may have negative effects on the pupil mental wellbeing as well as school behaviour. These are important consequences and should be assessed in better designed studies before these strategies can be recommended.

## Data Availability

The underlying data for this article consists of bibliographic references, which are included in the References section. Open Science Framework: Behaviour management in schools review https://doi.org/10.17605/OSF.IO/AJHGR ^25^ This project contains the following extended data: Supplementary data file- BMS review.docx PRISMA Flow Diagram PRISMA checklist PRISMA checklist and flowchart for ‘Disciplinary behaviour management strategies in schools and their impact on student psychosocial outcomes: A systematic review’. https://doi.org/10.17605/OSF.IO/AJHGR ^25^
