# Supplementary material for: Disciplinary behaviour management strategies in schools and their impact on student psychosocial outcomes: A systematic review
Source: NIHR Open Res. 2024 Jul 1;4:13. Originally published 2024 Mar 25. [Version 2] doi: 10.3310/nihropenres.13563.2 (PMC11320041; doi:10.3310/nihropenres.13563.2)
Supplement: Peer review history

# Reviewer report: Reviewer response for version 2

**DOI:** 10.3310/nihropenres.14788.r32270

**Published:** 2024-07-15

**Authors:** Nicholas A Gage

**Affiliations:** 1 Special Education Policy and Practice, WestEd, San Francisco, California, USA

The authors have addressed my concerns.

Are the rationale for, and objectives of, the Systematic Review clearly stated?

Yes

Is the statistical analysis and its interpretation appropriate?

Partly

Are sufficient details of the methods and analysis provided to allow replication by others?

Partly

Are the conclusions drawn adequately supported by the results presented in the review?

Yes

Reviewer Expertise:

systematic review, meta-analysis, and quantitate methods

I confirm that I have read this submission and believe that I have an appropriate level of expertise to confirm that it is of an acceptable scientific standard.

# Reviewer report: Reviewer response for version 1

**DOI:** 10.3310/nihropenres.14722.r31420

**Published:** 2024-04-29

**Authors:** Erica Gadsby

**Affiliations:** 1 Department of Health Sciences and Sport, University of Stirling, Stirling, Scotland, UK

This is a systematic review of disciplinary behaviour management strategies and their impact on student psychosocial outcomes. This is an area that is under-researched and of significant importance given the state of young people's mental health in the UK.

 The authors present a sound rationale, responding to the limited evidence on the intended impact, and concerns regarding unintended impact. 

 Researchers singled out psychosocial outcomes as a focus and conducted a systematic review to examine the impact of behaviour management on these outcomes.

 The question, in lay English was: How does school discipline affect pupil mental health and wellbeing?

 The introduction provided a good explanation of punitive approaches to school discipline, and alternative approaches (e.g. trauma informed, restorative, collaborative problem solving, etc.).

 The concern, that disciplinary strategies in school could be negatively impacting pupils' mental health, was raised in by young people within a consultation meeting in Bristol.

 Methods: The review was developed with input from young people - this involvement is described briefly in the public involvement section. It would be good to know whether this involvement was evaluated at all. The review had two secondary objectives: to assess whether the potential negative effects differed between different SES groups, and to see whether there was any evidence of the strategies positively impacting behaviour or academic outcomes.

 The focus are disciplinary measures of most concern was guided by young people - punitive measures, stopping short of permanent exclusion or expulsion. The review was limited to UK and OECD countries - justification that this does not include corporal punishment or other forms of restraint, which is reasonable. The population was young people aged 11 to 16, and the context mainstream school (not specialist schools). 

 Primary outcomes - any measures of mental health and wellbeing. 

 Secondary outcomes - academic and social outcomes.

 The search involved an information specialist and is well described. Grey literature was sought through targeted emails (via a network) - given none was found, it would be useful to reflect on whether the authors might have missed any grey literature that they might have found via another strategy? The process and conduct of the systematic review was as according to best practice. A narrative synthesis of the findings was the only thing possible, given the quality and type of data.

 In the description of included studies, the numbers don't add up. The paper states there are 14 studies in total: 10 from US, 2 from Australia and the UK (it's 2 from each, but this isn't very clear from the text), and 1 from Poland. That makes 15. I counted 9 from USA in table 1. What was the 10th?

 The study from Poland was in a primary school setting and the age of pupils is stated as 'not reported'. I'm wondering how this study fits the inclusion criteria?

 Discussion

 Evidence on the effects of school disciplinary strategies is scarce and of low quality. 

 There are caveats associated with the low quality evidence, but findings suggest a link between punitive disciplinary strategies and pupil mental health and wellbeing, and also social outcomes (social behaviour).

 Evidence of impact on educational attainment is limited (though that wasn't the focus of this review I guess? - have other reviews looked at this?).

 I found it a bit disappointing that the discussion didn't go into alternative disciplinary measures at all. The section ends: This can be remedied by enabling teachers use of evidence-based interventions that can reliably support pupil wellbeing. This could have usefully been followed up with a short discussion of what this might include.

Are the rationale for, and objectives of, the Systematic Review clearly stated?

Yes

Is the statistical analysis and its interpretation appropriate?

Not applicable

Are sufficient details of the methods and analysis provided to allow replication by others?

Yes

Are the conclusions drawn adequately supported by the results presented in the review?

Yes

Reviewer Expertise:

Health services and public health research focusing on social determinants of health

I confirm that I have read this submission and believe that I have an appropriate level of expertise to confirm that it is of an acceptable scientific standard.

# Response

**Published:** 2024-05-10

**Authors:** Sharea Ijaz

**Affiliations:** PHS, University of Bristol, Bristol, England, UK

**Thank you for your generous comments on and appreciation of our work. We have listed the queries in your review below and have responded to each.**

- The review was developed with input from young people - this involvement is described briefly in the public involvement section. It would be good to know whether this involvement was evaluated at all.

**The PPI for this project was not itself evaluated.** **However, some of the young PPI members have remained involved as stakeholders throughout the project and we presented our findings to them before finalizing this paper. Some of the young PPI members also became peer interviewers for the second part of the project, which is a qualitative study of pupil experience, and their experience as peer interviewers was evaluated qualitatively and this second part is currently being drafted as another paper: https://arc-w.nihr.ac.uk/research/projects/how-does-school-discipline-affect-pupil-mental-health-and-wellbeing/**

- Grey literature was sought through targeted emails (via a network) - given none was found, it would be useful to reflect on whether the authors might have missed any grey literature that they might have found via another strategy? The process and conduct of the systematic review was as according to best practice. A narrative synthesis of the findings was the only thing possible, given the quality and type of data.

**We used a pragmatic approach to find relevant literature in a field that has limited evidence.**

**We have added the below text to acknowledge the limitation of our grey search:**

**We found no studies from grey literature. Some of the databases we searched would have contained certain types of grey literature (e.g. conference abstracts, theses and dissertations). However, had we searched repositories of grey literature (e.g. OpenGrey or Overton (policy documents)), or the websites of international and regional education authorities, and government departments associated with our topic, we may have identified additional research published as monographs, reports, policy documents etc. It was difficult to translate our detailed search across to these grey literature sources, and we thought it more practical to talk to experts considering our limited resources.**

- In the description of included studies, the numbers don't add up. The paper states there are 14 studies in total: 10 from US, 2 from Australia and the UK (it's 2 from each, but this isn't very clear from the text), and 1 from Poland. That makes 15. I counted 9 from USA in table 1. What was the 10th?

**Thank you for spotting this. We are sorry for the error and have now corrected it: 9 studies from USA, 2 each from UK and Australia and 1 from Poland, equalling 14 in total.**

- The study from Poland was in a primary school setting and the age of pupils is stated as 'not reported'. I'm wondering how this study fits the inclusion criteria?

**This was an oversight from us. Thanks for pointing this out. “Primary school” in Poland lasts eight years (grades 1–8,  starting at age 6 or 7 and lasting until 14-15). This overlaps with primary and secondary school age in the UK. This specific study included 13–14-year-old students, which is compatible with secondary school age in the UK. We have now corrected and clarified this in the table.**

- Evidence of impact on educational attainment is limited (though that wasn't the focus of this review I guess? - have other reviews looked at this?).

**You are correct that this was a secondary outcome and not a focus of our review. In addition, the included study evidence was of very limited size and quality, so we did not expand on this.**

**Within our search we found two reviews on educational attainment outcome although they did not study our interventions of interest: **

1. **Craggs H, Kelly C. Adolescents' experiences of school belonging: A qualitative meta-synthesis. Journal of Youth Studies. 2018;21(10):1411-25. https://www.tandfonline.com/doi/full/10.1080/13676261.2018.1477125**
2. **Lee A, Gage NA. Updating and expanding systematic reviews and meta-analyses on the effects of school-wide positive behavior interventions and supports. Psychology in the Schools. 2020;57(5):783-804. https://onlinelibrary.wiley.com/doi/abs/10.1002/pits.22336**

**We found the following review outside of our search that included some evidence of link between academic outcomes and punitive disciplinary strategies:**

**Duarte CD, Moses C, Brown M, Kajeepeta S, Prins SJ, Scott J, Mujahid MS. Punitive school discipline as a mechanism of structural marginalization with implications for health inequity: A systematic review of quantitative studies in the health and social sciences literature. Ann N Y Acad Sci. 2023 Jan;1519(1):129-152. doi: 10.1111/nyas.14922. PMID: 36385456; https://pubmed.ncbi.nlm.nih.gov/36385456/**

**We have now added a sentence to expand on the evidence in reference to this review.**

  

- I found it a bit disappointing that the discussion didn't go into alternative disciplinary measures at all. The section ends: This can be remedied by enabling teachers use of evidence-based interventions that can reliably support pupil wellbeing. This could have usefully been followed up with a short discussion of what this might include.

**We have now added a sentence to indicate what these alternative strategies may be and that these can be useful:**

**There is growing evidence on trauma informed and restorative approaches for improving and managing disruptive behavior's in schools[52, 53] that are less likely to negatively impact pupil wellbeing.**

# Reviewer report: Reviewer response for version 1

**DOI:** 10.3310/nihropenres.14722.r31518

**Published:** 2024-04-25

**Authors:** Nicholas A Gage

**Affiliations:** 1 Special Education Policy and Practice, WestEd, San Francisco, California, USA

Thank you for the opportunity to review, Disciplinary behavior management strategies in schools and their impact on student psychosocial outcomes: A systematic review. This systematic review provides insights about the limited research and support for punitive disciplinary approaches in school. Overall, the manuscript is clearly written and the OSF materials provide more detail about the review. That being said, I do have a few concerns. First,

 at the start of the methods section, the authors note that they focus on secondary schools. There is no mention of secondary schools in the introduction/literature review. Consider noting differences between elementary and secondary school and discipline to justify why the study focuses on secondary schools. Second, there is not enough information or justification for why a meta-analysis was not conducted. I understand that a number of outcome domains have only one study, but as an example, there are five studies focused on suspensions and depression that could be meta-analyzed. In fact, a quantitative synthesis would be very helpful to better understand the results. Essentially, if the authors want to not provide a meta-analysis, a stronger rationale needs to be provided in the manuscript. Lastly, I found the exclusion reasons in the PRISMA figure to be lacking specificity. Consider revising with clearer language and reasons.

Are the rationale for, and objectives of, the Systematic Review clearly stated?

Yes

Is the statistical analysis and its interpretation appropriate?

Partly

Are sufficient details of the methods and analysis provided to allow replication by others?

Partly

Are the conclusions drawn adequately supported by the results presented in the review?

Yes

Reviewer Expertise:

more justification for only including secondary schools and not doing a meta-analysis are needed.

I confirm that I have read this submission and believe that I have an appropriate level of expertise to confirm that it is of an acceptable scientific standard, however I have significant reservations, as outlined above.

# Response

**Published:** 2024-05-10

**Authors:** Sharea Ijaz

**Affiliations:** PHS, University of Bristol, Bristol, England, UK

Thank you for the opportunity to review, Disciplinary behavior management strategies in schools and their impact on student psychosocial outcomes: A systematic review. This systematic review provides insights about the limited research and support for punitive disciplinary approaches in school. Overall, the manuscript is clearly written and the OSF materials provide more detail about the review. That being said, I do have a few concerns.

**Thank you for your feedback. We have made changes based on your comments and hope these are satisfactory**

- First, at the start of the methods section, the authors note that they focus on secondary schools. There is no mention of secondary schools in the introduction/literature review. Consider noting differences between elementary and secondary school and discipline to justify why the study focuses on secondary schools.

**We welcome your observation and have edited the introduction section to reflect the focus on secondary schools as follows:**

**End of para 1: These punitive approaches are commonplace in the western world [2, 3] and are more common in secondary schools in UK [4,5]**

**Para 4: This concern about the potential negative mental health impact on pupils was voiced by secondary school age young people in a public consultation meeting in Bristol (England) when collaboratively identifying research priorities.**

**Para 5: This review was then developed with input from these young people to investigate the existing evidence on the effects of punitive behaviour management strategies on mental health and wellbeing in secondary school age children and young people [22].**

  

- Second, there is not enough information or justification for why a meta-analysis was not conducted. I understand that a number of outcome domains have only one study, but as an example, there are five studies focused on suspensions and depression that could be meta-analyzed. In fact, a quantitative synthesis would be very helpful to better understand the results.

Essentially, if the authors want to not provide a meta-analysis, a stronger rationale needs to be provided in the manuscript.

**A meta-analysis was not conducted for the outcome of depression for suspensions as the 5 studies were very heterogeneous in not just populations (one study with 75% Black pupils, one study’s data from 1996), follow up times (no follow up in two studies, 1 year in two and 3 year in one study), outcome measures (dichotomous in 3 studies, continuous in 2) effect measures (odds ratios in 3 and regression coefficients in 2), comparisons (unspecified in three studies) but also study designs (one-time survey in two studies, repeated survey in two studies, and one controlled before-after study) and analyses (adjustment for confounders and numbers of analysed participants unclear in 3 studies).**

**We found the same for mixed strategies for depression outcome where the two studies (Chen 2021; Eyllon 2022) were very heterogeneous in study sample, follow up, and effect size.**

**We believe that pooling these disparate data from high risk of bias studies would not have changed our conclusions and recommendations but presented as a forest plot may give a false sense of validity to reader.**

**We have now edited the text to explain our choice in our synthesis methods:**

***We planned for a random effects meta-analysis if combinable data were available. However, a meta-analysis was not conducted as the studies were highly heterogeneous across populations, comparisons, follow up times, outcome measures, effect measures, and notably, study designs. The analyses presented were often unadjusted with numbers of analysed participants unclear. A narrative synthesis was therefore performed. Pooling these disparate data from high risk of bias studies in a meta-analysis would not have changed our conclusions and recommendations.***

- Lastly, I found the exclusion reasons in the PRISMA figure to be lacking specificity. Consider revising with clearer language and reasons.

**We had carried out screening of title/abstract and full texts in Rayyan and used these inbuilt labels in the platform to exclude and include studies. We have now revised the description in PRISMA to be more specific about the reasons.**
